# The Role and Efficiency of an AI-Powered Software in the Evaluation of Lower Limb Radiographs before and after Total Knee Arthroplasty

**DOI:** 10.3390/jcm12175498

**Published:** 2023-08-24

**Authors:** Stefano Pagano, Karolina Müller, Julia Götz, Jan Reinhard, Melanie Schindler, Joachim Grifka, Günther Maderbacher

**Affiliations:** 1 Department of Orthopedic Surgery, University of Regensburg, Asklepios Klinikum Bad Abbach, 93077 Bad Abbach, Germany; 2 Center for Clinical Studies, University of Regensburg, 93053 Regensburg, Germany

**Keywords:** artificial intelligence, medical imaging analysis, musculoskeletal radiology, total knee arthroplasty, lower limb radiography analysis, software efficiency

## Abstract

The rapid evolution of artificial intelligence (AI) in medical imaging analysis has significantly impacted musculoskeletal radiology, offering enhanced accuracy and speed in radiograph evaluations. The potential of AI in clinical settings, however, remains underexplored. This research investigates the efficiency of a commercial AI tool in analyzing radiographs of patients who have undergone total knee arthroplasty. The study retrospectively analyzed 200 radiographs from 100 patients, comparing AI software measurements to expert assessments. Assessed parameters included axial alignments (MAD, AMA), femoral and tibial angles (mLPFA, mLDFA, mMPTA, mLDTA), and other key measurements including JLCA, HKA, and Mikulicz line. The tool demonstrated good to excellent agreement with expert metrics (ICC = 0.78–1.00), analyzed radiographs twice as fast (*p* < 0.001), yet struggled with accuracy for the JLCA (ICC = 0.79, 95% CI = 0.72–0.84), the Mikulicz line (ICC = 0.78, 95% CI = 0.32–0.90), and if patients had a body mass index higher than 30 kg/m^2^ (*p* < 0.001). It also failed to analyze 45 (22.5%) radiographs, potentially due to image overlay or unique patient characteristics. These findings underscore the AI software’s potential in musculoskeletal radiology but also highlight the necessity for further development for effective utilization in diverse clinical scenarios. Subsequent studies should explore the integration of AI tools in routine clinical practice and their impact on patient care.

## 1. Introduction

The critical assessment of knee alignment, leg length discrepancies, and other associated anatomical aspects intrinsic to the lower extremities requires a comprehensive analysis of radiographic images [1,2,3,4,5].

These assessments play a crucial role in surgical planning and postoperative evaluation, particularly in leg alignment correction procedures [6]. The traditional approach involves the use of interactive software applications, which can lead to inconsistencies in measurements and require significant time from the physician [7].

Fully automated measurements offer a solution to these challenges, especially the inaccuracy and extended time demands of traditional methods. They not only optimize the process but also enhance accuracy and repeatability compared to current methods. Computer-aided measurements have been progressively incorporated into radiological practices to improve the precision and reproducibility of measurements, surpassing the challenges of manual efforts. Such computerized processes are useful in diverse medical imaging areas such as cardiovascular, musculoskeletal, and neurological imaging [8,9,10,11].

The broader implications of employing such advanced techniques in clinical practice can potentially revolutionize patient care by minimizing human error, improving surgical planning, and therefore enhancing postoperative outcomes [11,12,13,14].

Orthopedic radiology has significantly profited from artificial intelligence (AI). It demonstrates potential in reducing measurement errors, increasing efficiency, and improving repeatability, particularly in evaluating the lower extremities [12,13].

With traditional radiographic methods often yielding inconsistent and non-standardized measurements, the need for reliable and reproducible automated measurement tools is increasingly evident [14].

Based on the above, our research hypothesis suggests that an AI-powered software could achieve consistency and accuracy comparable to physicians in evaluating lower limb alignments.

We conducted a study to assess the concordance between the software LAMA (Version 1.13.16, September 2022, IB Lab GmbH, Vienna, Austria) and two orthopedic specialists in estimating various lower extremity metrics.

LAMA, which stands for leg angle measurement assistant, provides an automated approach to assess angle and length measurements on lower extremity radiographs, subsequently generating graphical annotations on the respective DICOM images. It uses a U-Net-based convolutional neural network designed for biomedical image analysis and has been rigorously trained on more than 15,000 radiographs from different studies. This software delivers fully automated measurements on these radiographs, ensuring rapid results without the requirement for other interactive applications [15,16,17].

## 2. Materials and Methods

### 2.1. Objective of the Study

This study assessed the efficacy of LAMA, a computer-aided detection (CADe) system, in identifying lower limb alignment using anteroposterior (AP) standing lower extremity radiographs. Automated calculations derived from the software were compared against a clinical reference benchmark comprising evaluations from two orthopedics.

### 2.2. Study Data

**Figure 1:**
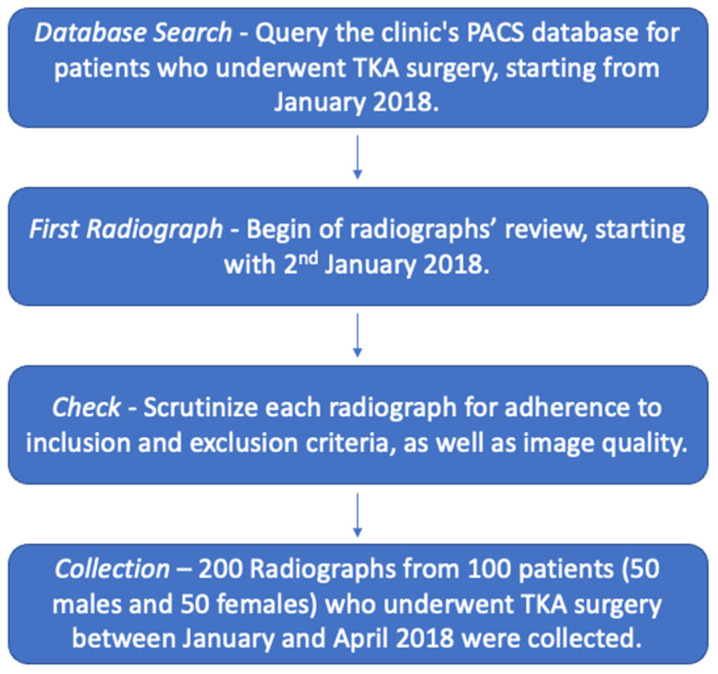
Flowchart illustrating the patient selection process for 200 radiographs from the clinic’s database.

In this study, 200 archived radiographs of 100 patients, equally distributed by gender, who underwent total knee arthroplasty (TKA) surgery in the last five years at our institution (Department of Orthopedic Surgery of the University of Regensburg, Germany), and who had imaging before and after the procedure were retrospectively evaluated (Figure 1). Radiographs were selected pseudonymously from our clinic’s PACS database.

The inclusion criteria for the radiographs were as follows:

- The patient was at least 18 years of age.
- The patient had undergone TKA surgery within the past five years.
- The TKA surgery was a primary procedure due to gonarthrosis.
- The patient was referred for both pre- and post-surgical full-length AP standing lower extremity imaging.
- The digital X-ray image was acquired within the last five years.

Radiographs were excluded under the following cases:

- The patient had fractures at the time of imaging.
- There was evidence of implant failure in the postoperative X-ray.
- Visible knee implants were present presurgically (such as TKA, unicondylar knee arthroplasty (UKA), high tibia osteotomy (HTO), surgical screws, plates).
- Image quality issues prevented the identification of markers necessary for measurements.
- The surgical indication for TKA was for reasons other than gonarthrosis.

Parameters measured by the LAMA software and two orthopedics included the mechanical axis deviation (MAD), mechanical lateral proximal femoral angle (mLPFA), anatomical mechanical angle (AMA), mechanical lateral distal femoral angle (mLDFA), joint-line convergence angle (JLCA), mechanical medial proximal tibia angle (mMPTA), mechanical lateral distal tibia angle (mLDTA), hip-knee-ankle angle (HKA), and mechanical axis length (Mikulicz line). The presence of leg axis deviations from neutral, classified as either varus or valgus, was also determined.

Furthermore, the time required to measure each radiograph was recorded for both the AI and the orthopedics. These parameters allow the assessment of time efficiency as well as agreement (inter-rater and intra-rater reliability).

Additional data, such as patient demographics and DICOM metadata, were collected from medical records.

### 2.3. Evaluation of Radiographs

The evaluation of the radiographs was performed by AI software (LAMA, Version 1.13.16, September 2022, IB Lab GmbH, Vienna, Austria) and two orthopedic specialists: a resident doctor with three years of experience (Rater 1) and a senior surgeon with a decade of experience (Rater 2). Both raters independently provided the same measurements from the identical radiographs, without knowledge of the software’s estimates, to assess inter-rater reliability.

**Figure 2:**
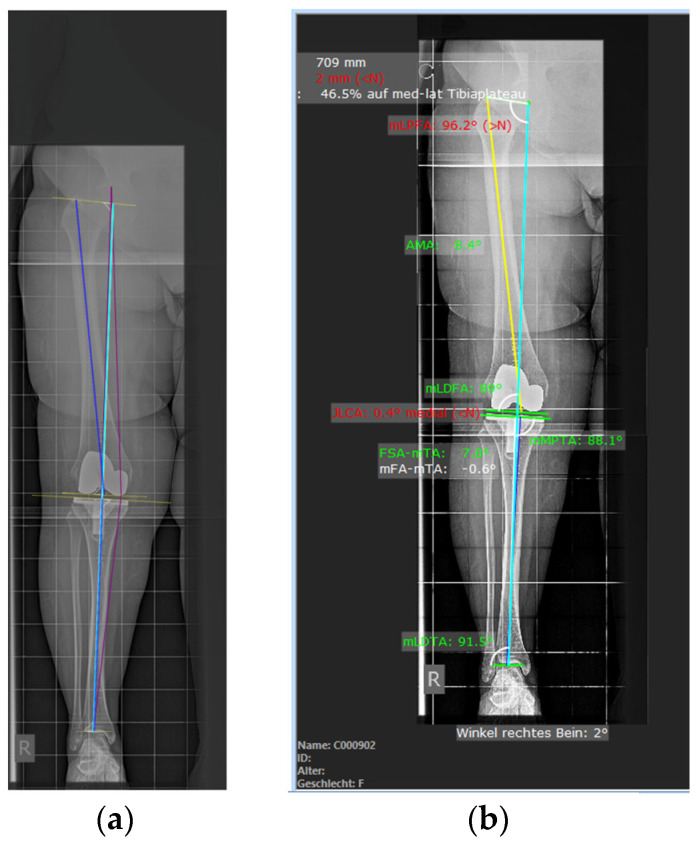
Comparison between the visual output of measurements obtained from the software on the left (**a**) and those performed by two orthopedics using the mediCAD software (**b**). The planning software at our institution provides a more extensive array of morphometric variables. However, for the purposes of this study, only the same variables measured through both software platforms were considered.

The junior orthopedic (Rater 1) also performed a second read after 4 weeks to assess intra-rater reliability. The orthopedics utilized the current clinical workflow software mediCAD (Version 6.5, mediCAD Hectec GmbH, Altdorf, Germany) for their evaluations. The software was executed on a 64-bit computer with a Windows 11 operating system, powered by an Intel Core i5-6500 processor running at 3.20 GHz, along with 8 GB of RAM (Figure 2).

In our clinic, as a standard procedure, only the preoperative radiographs include a graduated sphere. This scaling sphere, with a known diameter, is essential for calibration, serving as a reference point to provide accurate length measurements on the radiographic images. To address the measurement challenges in postoperative radiographs where a graduated sphere was not present, the orthopedics implemented a distinctive approach. Using the preoperative radiographs, the orthopedics measured the diameter of a known reference object, such as the femoral head or hip prosthesis head, using the graduated sphere for accurate scaling. Subsequently, this reference diameter was employed for postoperative radiographs as a scaling proxy.

### 2.4. Statistical Analysis

Descriptive analyses of patient characteristics and number of measured parameters in radiographs included absolute (n) and percentual (%) frequencies for categorical variables, and mean (m) and standard deviation (SD) as well as minimum and maximum for continuous variables.

To assess whether the failure of measuring parameters in radiographs by the software was related to the body mass index (BMI), a U-test was used. Therefore, measurements were dichotomized into success (more than half of the nine parameters could be measured) and failure (none of the nine parameters could be measured), and BMI was categorized as normal weight (BMI 18.5–24.9), overweight (BMI 25.0–29.9), and obesity (BMI > 30).

To assess the time efficacy, the mean time (in seconds) required to measure the nine parameters in the radiographs was compared between the software and each rater, between Rater 1 and Rater 2, and between two measurements (four weeks apart) of Rater 1 by paired *t*-tests.

Furthermore, we investigated the agreement between the orthopedics’ and the software’s measurements.

In a first step, measurements of all three raters (without distinction between pre- and postoperative) were assessed by intraclass correlation coefficients (ICC, two-way mixed effects model, mean of raters) for continuous parameters and Fleiss’ kappa for nominal parameters (inter-rater reliability) [18,19]. As the postoperative radiographs did not include a graduated sphere, ICC and Fleiss’ kappa were separately calculated for pre- and postoperative radiographs. Values over 0.90 indicate excellent agreement, values between 0.75 and 0.90 indicate good agreement, values between 0.50 and 0.75 indicate moderate agreement, and values less than 0.50 indicate poor agreement.

As sensitive analyses, inter-rater reliability analyses (overall ICC and Cohen’s kappa) were repeated for direct comparisons between Rater 1 and Rater 2 as well as between Rater 1 and the software, and Rater 2 and the software. Sensitivity analyses were conducted due to two reasons. First, clinicians were able to estimate each parameter in each radiograph and the software could not. Second, clinicians had different levels of experience. Additionally, clinically relevant differences between estimates of the raters and the software were defined by a deviation of more than ±2° for angle measurements and more than ±5 mm for length measurements.

These criteria were chosen based on two pertinent sources of research. Our approach for angle measurements adopts a more conservative strategy compared to the conclusions of the study conducted by Parrate et al. [20]. Their investigation into TKA implant durability, based on the mechanical axis, established an acceptable alignment threshold of 0° ± 3°. In contrast, our study aims to account for even minor deviations that could potentially influence preoperative planning by implementing a more rigorous benchmark of 2°. This more stringent standard aligns with the clinically significant value utilized in two prior investigations on the precision of the LAMA software [15,16]. Consequently, this choice facilitates direct comparisons with the results of those previous studies.

Regarding length measurements, our second criterion is in line with findings from Knutson’s review, suggesting that 90% of the population exhibits an almost negligible difference in anatomic leg length, averaging approximately 5.2 mm [21]. By setting our threshold at 5 mm, we avoid overstating minor variations that typically have no clinical significance. Notably, this target was also referenced by the aforementioned studies. Thus, we have maintained this benchmark, ensuring consistency with the current literature.

Moreover, we employed Bland–Altman plots to estimate the degree of agreement for three primary knee alignment indicators—HKA, MAD, and JLCA. These indicators were specifically chosen due to their essential role in evaluating overall limb alignment, the positioning of the mechanical axis, and the joint convergence angle. All these factors significantly influence the outcome of total knee arthroplasty, emphasizing the importance of precise measurement and strong agreement between different measurement methods [22].

Lastly, the agreement of two measurements made by Rater 1 (intra-rater reliability: ICC and Cohen’s kappa) and clinically relevant differences were assessed.

SPSS software (version 29, IBM) was used for the statistical analysis. The level of significance was defined at two-sided ≤ 0.050. This analysis was of an exploratory nature, thus no adjustments for multiple testing were made.

This research adhered to the guiding principles outlined in the Declaration of Helsinki. The Institutional Review Board of the University of Regensburg (Germany) granted ethical approval (Approval number: 20-1927-101).

## 3. Results

A total of 200 radiographs of 100 patients pre- and post-TKA surgery were evaluated.

### 3.1. Patient Characteristics

The age of the patients varied from 46 to 84 years, with an average age of 66.8 years (SD = 8.2 years). The sex was evenly distributed (n = 50 male, n = 50 female). The average BMI was 29.8 kg/m^2^ (SD = 5.1 kg/m^2^) ranging from 20.5 to 44.8 kg/m^2^. Normal weight was recorded for 15 (15%), overweight for 39 (39%), and obesity for 46 (46%) patients.

### 3.2. Measurement of Radiograph Parameters

For each of the 200 radiographs, nine parameters (MAD, mLPFA, AMA, mLDFA, JLCA, mMPTA, mLDTA, HKA, Mikulicz line) along with the type of leg axis deviation were planned to be measured by the LAMA software and two orthopedics. The medical evaluators could measure all parameters in each radiograph. The LAMA software could only assess all nine parameters in 136 (68%) radiographs. In 45 (22.5%) radiographs, none of the nine parameters could be measured (n = 25 preoperative, n = 20 postoperative). Partial measurement was possible in 19 (9.5%) radiographs: seven parameters in 4 (2%) radiographs and eight parameters in 15 (7.5%) radiographs, while five times MAD, five times JLCA, and 13 times the Mikulicz line could not be measured. For 11 patients, both pre- and postoperative radiographs could not be analyzed by LAMA (Table 1).

In the subgroup of radiographs that the software failed to recognize, 68.9% were from patients classified as obese. In contrast, of the radiographs that were successfully analyzed by the software, 60.7% were from patients with either normal weight or overweight (Table 2).

**Figure 3:**
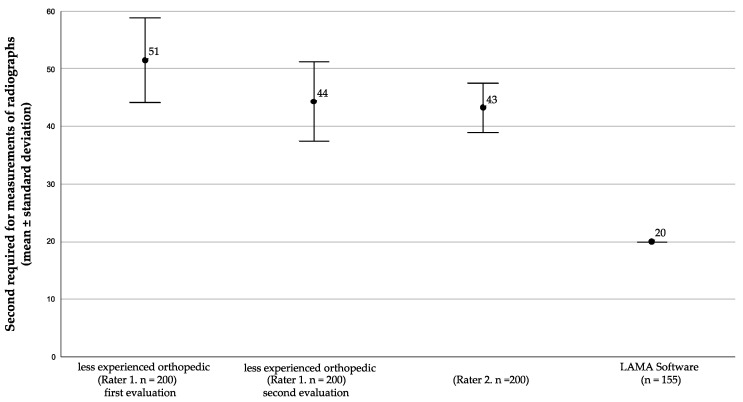
Average time required for image evaluations. In this figure, the average time taken by Rater 1 (for both measurements), Rater 2, and the software to evaluate the radiographs are shown. For the software, the time was calculated by dividing the total processing time by the number of analyzed radiographs.

The LAMA software showed superior time efficiency. The measurement of one radiograph required 20 s by the software and was more than twice as fast as the orthopedics’ measurements (*p* values < 0.001, n = 155). The less experienced orthopedic (Rater 1) needed on average 51.6 s (SD = 7.7 s) per radiograph for the initial evaluation and 44.9 s (SD = 7.4 s) for the secondary evaluation. In comparison, the more experienced orthopedic (Rater 2) required on average 43.2 s (SD = 4.2 s) (Figure 3).

### 3.3. Agreement between Measurements

Overall, inter-rater reliability of the LAMA software and expert measurements ranged from 0.78 to 1.00, indicating an overall good to excellent level of agreement (Table 3). However, the ICC 95% confidence interval (CI) of the Mikulicz line estimates varied widely from 0.32 to 0.90.

Separate analyses of pre- and postoperative radiographs (Table 3) showed that the wide range of ICC (95% CI = 0.03–0.89) of the Mikulicz line estimates was attributable to the postoperative radiographs with missing scaling spheres. Moreover, separate analyses indicated a poor level of agreement in JCLA (preoperative ICC = 0.49, 95% CI = 0.25–0.66, postoperative ICC = 0.47, 95% CI = 0.23–0.65). All other parameters showed a moderate to excellent level of agreement.

In seven out of 75 preoperative radiographs, the software was not able to detect the scaling sphere. This failure had no influence on the inter-rater reliability.

In a second step, direct comparisons of radiograph measurements (overall) between each rater (Rater 1, Rater 2, software) were conducted. As for the inter-rater reliability among expert raters, high agreement rates were observed ranging from 0.90 to 1.00, except for AMA values where the agreement was less substantial (ICC = 0.61, 95% CI= 0.48–0.71) (Table 4). When comparing the measurements provided by the software with those taken by each orthopedic, substantial variability in reliability coefficients of the Mikulicz line and JCLA estimates were observed, indicating poor agreement. AMA showed a moderate level of agreement between Rater 1 and the software (ICC = 0.69, 95% CI = 0.58–0.78), but an excellent level of agreement between Rater 2 and the software (ICC = 0.91, 95% CI = 0.85–0.94). All other parameters showed comparable agreement from moderate to excellent levels (Table 4).

We noted some clinically significant discrepancies between the AI measurements and those from the evaluators. In the cases of MAD, AMA, and HKA, the differences were relatively infrequent. Specifically, the differences between Rater 1 and the AI appeared in MAD measurements in 8 out of 150 cases (5.3%), in AMA measurements in 14 out of 155 cases (9.0%), and in HKA measurements in 5 out of 155 cases (3.2%). When comparing Rater 2 with the AI, these differences were found in MAD measurements in 10 out of 150 cases (6.7%), in AMA measurements in 1 out of 155 cases (0.6%), and in HKA measurements in 7 out of 155 cases (4.5%). However, in the case of the Mikulicz line, the discrepancies were notably more prevalent. In this instance, the software generated values that diverged more than ±5 mm from those of the two physicians in 135 out of 142 cases (95.1%) (Table 5).

In the intra-rater reliability, moderate agreement was observed in measurements regarding AMA (ICC = 0.67, 95% CI 0.57–0.75) (Table 6).

**Figure 4:**
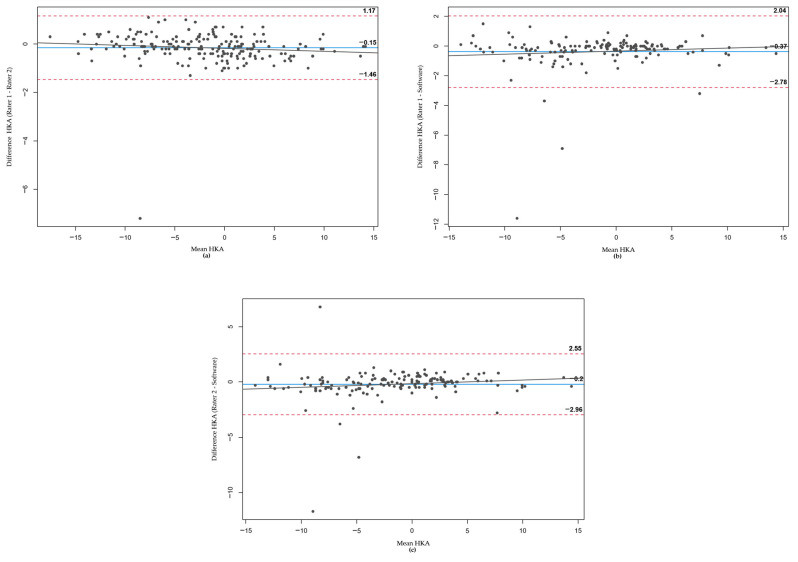
Bland–Altman plots for HKA measurements. (**a**–**c**) represent the comparisons between Rater 1 and Rater 2, Rater 1 and the software, and Rater 2 and the software, respectively. Each plot features the mean values on the X-axis and the differences on the Y-axis, with the pointed lines indicating the 95% confidence interval.

**Figure 5:**
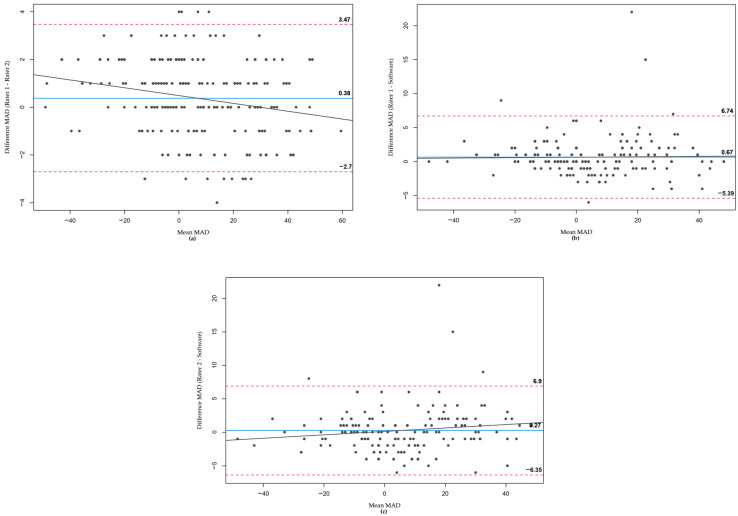
Bland–Altman plots for MAD measurements. (**a**) Rater 1 vs. Rater 2, (**b**) Rater 1 vs. software, (**c**) Rater 2 vs. software.

**Figure 6:**
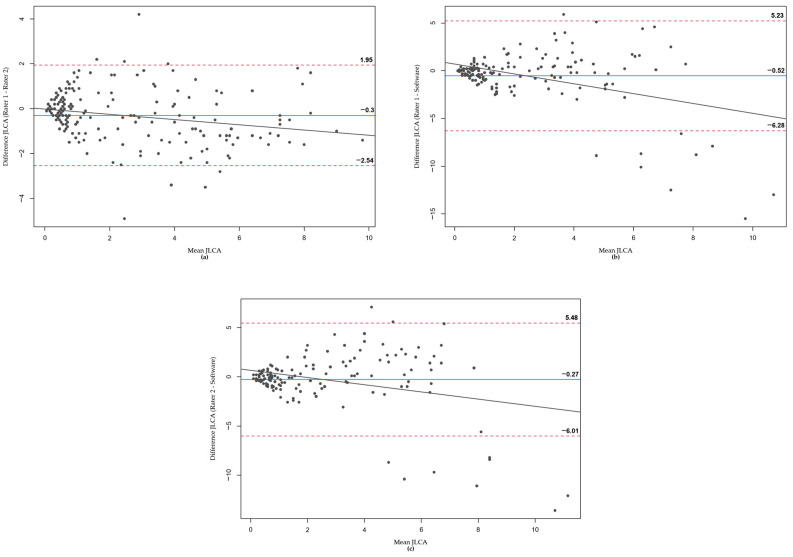
Bland–Altman plots for JLCA measurements. (**a**) Rater 1 vs. Rater 2, (**b**) Rater 1 vs. software, (**c**) Rater 2 vs. software.

In the Bland–Altman plots constructed for HKA (Figure 4), MAD (Figure 5), and JLCA (Figure 6), differences between the two raters and the software were plotted against their mean values. For each parameter, comparisons were made between Rater 1 and Rater 2 (Figure 4a, Figure 5a and Figure 6a), Rater 1 and the software (Figure 4b, Figure 5b and Figure 6b), and Rater 2 and the software (Figure 4c, Figure 5c and Figure 6c).

Across all parameters and comparisons, the majority of values fell within the 95% confidence interval. In the few instances where the software’s measurements deviated significantly from those of the two orthopedics, these outliers are visible in the respective plots.

## 4. Discussion

The findings of our study suggest that IB Lab’s LAMA software serves as a reliable tool in assessing lower limb alignment, employing AP standing lower extremity radiographs, regardless of the presence or absence of arthroplasty implants.

Since its development, this software has caught the interest of the scientific community, leading to its examination, at the time of this writing, in two research papers. For instance, Simon et al. were among the first to evaluate the software’s capability [15]. The authors examined the precision of the software on a collection of 295 preoperative standing long-leg anteroposterior radiographs of patients undergoing total knee arthroplasty surgery. Their study indicated a success rate of 98.0% of LAMA recognizing the X-rays.

Comparatively, our investigation, despite exhibiting a modest recognition rate of 77.5%, unveiled a significant agreement between AI-derived measurements and those assessed by two different experienced orthopedic professionals. Echoing the opinions expressed by Simon et al., who emphasized that minor adjustments in landmark setting can significantly influence angle measurements (such as JLCA, mLDTA, mLPFA, and mMPTA) and drew attention to the absence of standardization concerning the several reference points, our study too acknowledged the analogous conclusion. We observed a slightly lesser agreement between the software’s readings and expert evaluations, particularly for the JLCA values in both preoperative and postoperative scenarios. Occasionally, the software inaccurately positioned the axes passing through the bases of the femoral condyles and/or the tibial plateau in its measurement protocols, which could have contributed to the less-than-optimal agreement for this specific measurement. Still, this discrepancy was even apparent in the intra-rater reliability.

Our findings indicate inconsistencies in measurements, primarily due to the subjective nature of landmark selection. Furthermore, osteophytes, particularly those located at the edges of the joint, introduce a significant degree of variation when pinpointing the knee axes’ center. In this context, Bowman et al. [23] offer a valuable insight. The severity of anatomical deformities may introduce an additional layer of variability to the measurements. Interestingly, despite these challenges, their study also underscores the strength of manual methods, highlighting that they offer substantial reliability across different levels of experience.

While manual methods possess inherent advantages, the potential for human error and subjective variability in measurements suggests the value of automated systems. Such methods could offer a standardized, fixed decision model, minimizing subjective variability and providing enhanced accuracy and reliability.

This stance is supported by the findings of Simon et al., which demonstrated a high level of agreement—99.6% for lengths and 100% for uncalibrated lengths—in repeated measurements using the LAMA software.

Another advantage of automated systems is the considerable time-saving aspect. In our study, the software measured at twice the speed of medical evaluators. However, it is relevant to acknowledge the disparities in efficiency noted between the study by Simon et al., which took 62 s per radiograph, and our own, where the mean processing time was 20 s. These variations, however, may reflect the differing computational capacities of the hardware deployed in each study or even enhancements in the software, given that our study used a more recent version (1.13.16 against 1.03.17 used in the previous study).

Another investigation, led by Schwarz et al., probed the efficacy of the IB Lab LAMA software on 200 weight-bearing lower extremity radiographs obtained from 172 patients after a total knee arthroplasty [16]. They observed a high correlation between AI and manual measurements (ICC > 0.97). Though our study yielded slightly lower ICC values (ranging from 0.78 to 1.00), it nevertheless demonstrated moderate to excellent agreement. The slightly lower values in our study could be related to the more extensive confidence intervals for the Mikulicz line measurement. This discrepancy can be attributed to the routine inclusion of the scaling sphere in only preoperative radiographic images at our institution. Its absence in postoperative images may have introduced higher inaccuracy in length measurements. Utilizing preoperative radiographs, the orthopedics determined the diameter of a known reference, subsequently using it as a scaling reference for postoperative images. This approach accentuates human adaptability in varying image conditions, a trait AI-based systems like LAMA still need to refine [24].

The LAMA software demonstrated an increased rate of unsuccessful image analyses in our dataset, with a failure rate of 22.5% primarily due to landmark recognition challenges. This contrasts plainly with the 2% and 4% failure rates reported by Simon et al. and Schwarz et al. This divergence could arise from our study’s diverse patient cohort and the variety of joint implants in our X-ray images.

Furthermore, all radiographs in our study featured a superimposed raster, whereas the aforementioned investigations used images without such graphical elements. This grid could introduce complexities to image analysis, potentially leading the software to misinterpret these lines as anatomical landmarks. This observation suggests a need for further refining the LAMA algorithm or implementing a preprocessing step to optimize images for analysis.

Additionally, our study identified challenges with the LAMA software when processing radiographic images from patients with a BMI exceeding 30 kg/m^2^. The excessive adipose tissue associated with higher BMI can result in denser radiographic projections, thereby obscuring the outlines of critical anatomical landmarks, which could impact not only AI-based tools like LAMA but also potentially confound manual image interpretation, underlining the considerable impact of patient demographics and physical attributes on the precision of the measurements.

Our study, although insightful, has several limitations. We could not definitively pinpoint the reasons behind the software’s failure to analyze certain radiographs due to the inherent ‘black box’ nature of AI algorithms [25]. Moreover, the heterogeneity of our large patient cohort, with inclusive criteria and the presence of varying degrees of arthrosis, may have contributed to our lower ICC values relative to earlier studies.

Another limitation to highlight is the evaluation of inter-rater reliability between only two clinicians of differing expertise levels. Moreover, the intra-rater reliability was assessed only by the less experienced orthopedic. A broader team of evaluators might have provided deeper insights into the actual variability in measurements between physicians.

There were also several limitations concerning the radiographs used. All the DICOM radiographs had a raster overlay, which could not be removed due to its integration within the source file. The scaling sphere, which could have enabled the software to achieve more accurate length measurements, was absent in the postoperative radiographs. The presence of a hip prosthesis could also have interfered with the software’s processing, a factor not investigated in this study.

All these aforementioned potential interferences with the software’s capability to accurately identify landmarks and joint outlines might have further contributed to the lower ICC values observed in our study.

## 5. Conclusions

Our research, complemented by studies from Simon et al. and Schwarz et al., highlights both the potential advantages and challenges of using AI software like LAMA in musculoskeletal radiology.

The inter-rater reliability between the software and orthopedic specialists demonstrated excellent agreement for the assessment of parameters such as MAD, mLPFA, mLDTA, and HKA, where the ICC exceeded 0.90. In contrast, the evaluation of AMA, mLDFA, mMPTA, JLCA, and the Mikulicz line yielded a marginally lower agreement, though the ICC still surpassed 0.75.

However, the inter-rater reliability in measuring JLCA and the Mikulicz line fell short of the expected standard. This limitation is evident when examining the broader 95% CI range for these parameters. Notably, the lower bound of the CI for both JLCA and the Mikulicz line dipped below 0.75, suggesting potential inconsistencies and reduced reliability in certain scenarios.

While these AI-powered solutions demonstrate remarkable accuracy and efficiency, they also face challenges, underlining the ongoing need for refinement, especially in varied patient populations and settings [24,26,27,28]. Continued collaboration between clinicians and software developers is essential to adapt these technologies to meet the evolving demands of orthopedic practice. Future research should explore the integration of such tools into the clinical routine and assess their impact on enhancing patient care.

## Figures and Tables

**Table 1:** Correctly measured parameters of pre- and postoperative radiographic images by the software.

		Post-Operative			Total
	Number of Measured Parameters	0	8	9	
**Pre-operative**	**0**	11	2	12	25
	**7**	0	4	0	4
	**8**	0	2	2	4
	**9**	9	3	55	67
	**Total**	20	11	69	100

**Table 2:** Comparison of BMI between successful and unsuccessful measurements of parameters in radiographs using the software.

			Body Mass Index			Total
			18.5–24.9	25.0–29.9	>30	
			Normal Weight	Overweight	Obesity	
**Evaluation of radiographs**	**Failure**	n	2	12	31	45
	**(no parameter was measured)**	%	4.40%	26.70%	68.90%	100.00%
	**Success**	n	28	66	61	155
	**(more than half of the parameters were measured)**	%	18.10%	42.60%	39.40%	100.00%
**Total**		n	30	78	92	200
		%	15.00%	39.00%	46.00%	100.00%
*p* < 0.001						

**Table 3:** Inter-rater reliability of the two experts and the algorithmic system. The following measured parameters were compared: mechanical axis deviation (MAD), mechanical lateral proximal femoral angle (mLPFA), anatomical mechanical angle (AMA), mechanical lateral distal femoral angle (mLDFA), joint-line convergence angle (JLCA), mechanical medial proximal tibia angle (mMPTA), mechanical lateral distal tibia angle (mLDTA), hip-knee-ankle angle (HKA), mechanical axis length (Mikulicz line) and leg axis (varus, valgus).

	Inter-Rater Reliability-Rater 1 vs. Rater 2 vs. Software											
	Total				Pre-Operative				Post-Operative			
	n	ICC	95% CI		n	ICC	95% CI		n	ICC	95% CI	
**MAD**	150	1	0.99	1	70	1	1	1	80	0.99	0.99	1
**mLPFA**	155	0.93	0.91	0.95	75	0.93	0.89	0.95	80	0.94	0.91	0.96
**AMA**	155	0.81	0.75	0.86	75	0.89	0.83	0.92	80	0.73	0.61	0.82
**mLDFA**	155	0.87	0.85	0.91	75	0.83	0.75	0.89	80	0.98	0.96	0.98
**JLCA**	150	0.79	0.72	0.84	74	0.49	0.25	0.66	76	0.47	0.23	0.65
**mMPTA**	155	0.86	0.82	0.89	75	0.82	0.73	0.88	80	0.93	0.88	0.96
**mLDTA**	155	0.95	0.92	0.96	75	0.95	0.92	0.97	80	0.94	0.91	0.97
**HKA**	155	0.99	0.99	0.99	75	0.99	0.99	0.99	80	1	0.99	1
**Mikulicz line**	142	0.78	0.32	0.9	69	0.95	0.83	0.98	73	0.69	0.03	0.89
	n	Kappa	95% CI		n	Kappa	95% CI		n	Kappa	95% CI	
**Leg Axis**	153	0.92	0.83	1.01	75	0.93	0.8	1.06	78	0.9	0.77	1.02

ICC: Intraclass Correlation Coefficient, CI: Confidence Interval.

**Table 4:** Inter-rater reliability among raters and the software: This table depicts the inter-rater reliability between each of the two orthopedics raters and the software.

	Inter-Rater Reliability											
	Rater 1 vs. Software				Rater 2 vs. Software				Rater 1 vs. Rater 2			
	n	ICC	95% CI		n	ICC	95% CI		n	ICC	95% CI	
**MAD**	150	0.99	0.99	0.99	150	0.99	0.99	0.99	200	1	1	1
**mLPFA**	155	0.87	0.79	0.91	155	0.88	0.83	0.91	200	0.96	0.94	0.97
**AMA**	155	0.69	0.58	0.78	155	0.91	0.85	0.94	200	0.61	0.48	0.71
**mLDFA**	155	0.81	0.74	0.86	155	0.77	0.69	0.83	200	0.97	0.96	0.98
**JLCA**	150	0.58	0.43	0.7	150	0.63	0.49	0.73	200	0.94	0.92	0.96
**mMPTA**	155	0.75	0.65	0.82	155	0.71	0.6	0.79	200	0.97	0.95	0.97
**mLDTA**	155	0.9	0.83	0.94	155	0.89	0.8	0.94	200	0.97	0.96	0.98
**HKA**	155	0.99	0.98	0.99	155	0.98	0.98	0.99	200	1	1	1
**Mikulicz line**	142	0.59	−0.19	0.83	142	0.61	−0.18	0.84	200	1	1	1
	n	Kappa	95% CI		n	Kappa	95% CI		n	Kappa	95% CI	
**Leg Axis**	153	0.93	0.87	0.99	153	0.92	0.86	0.98	200	0.9	0.83	0.96

ICC: Intraclass Correlation Coefficient, CI: Confidence Interval.

**Table 5:** Clinically relevant differences in parameter estimates and leg axis definition: Clinical relevancy was determined by a divergence in measurements of more than 2° for angles or over 5 mm for lengths.

	Rater 1 vs. Software				Rater 2 vs. Software				Rater 1 vs. Rater 2			
	Clinically Relevant Difference (>2°, >5 mm, >0.5 cm)											
	n	Yes	No	Discrepancy (%)	n	Yes	No	Discrepancy (%)	n	Yes	No	Discrepancy (%)
**MAD**	150	8	142	5.3	150	10	140	6.7	200	0	200	0
**mLPFA**	155	65	90	41.9	155	69	86	44.5	200	73	127	36.5
**AMA**	155	14	141	9	155	1	154	0.6	200	35	165	17.5
**mLDFA**	155	20	135	12.9	155	22	133	14.2	200	4	196	2
**JLCA**	150	29	121	19.3	150	34	116	22.7	200	15	185	7.5
**mMPTA**	155	24	131	15.5	155	36	119	23.2	200	7	193	3.5
**mLDTA**	155	20	105	12.9	155	58	97	37.4	200	32	168	16
**HKA**	155	5	150	3.2	155	7	148	4.5	200	1	199	0.5
**Mikulicz line**	142	135	7	95.1	142	135	7	95.1	200	10	190	5
	**Varus/Valgus Deviation**											
	n	No Agreement	Agreement	Discrepancy (%)	n	No Agreement	Agreement	Discrepancy (%)	n	No Agreement	Agreement	Discrepancy (%)
**Leg Axis**	153	5	148	3.3	153	6	147	3.9	200	10	190	5

**Table 6:** Intra-rater reliability of Rater 1: The measurements of the same radiographs by Rater 1, taken four weeks apart, are compared to assess intra-rater reliability.

	Intra-Rater Reliability-Rater 1 First vs. Second Measurement				Clinically Relevant Difference	
					(>2°, >5 mm, >0.5 cm)	
	n	ICC	95% CI		Yes	No
**MAD**	200	1	1	1	0	200
**mLPFA**	200	0.97	0.96	0.98	45	155
**AMA**	200	0.67	0.57	0.75	48	152
**mLDFA**	200	0.97	0.97	0.98	5	195
**JLCA**	200	0.95	0.94	0.97	10	190
**mMPTA**	200	0.98	0.98	0.99	5	195
**mLDTA**	200	0.97	0.96	0.98	25	175
**HKA**	200	1	1	1	1	198
**Mikulicz line**	200	0.97	0.96	0.98	108	92
	n	Kappa	95% CI		No Agreement	Agreement
**Leg Axis**	200	0.95	0.9	0.99	5	195

ICC: Intraclass Correlation Coefficient, CI: Confidence Interval.

## Data Availability

The data presented in this study are available in the article.
